# The Importance of Parents for Childhood and Adolescent Obesity Prevention: Should We Pay More Attention to Automatic Processes and Parental Stress?

**DOI:** 10.3390/nu13093185

**Published:** 2021-09-13

**Authors:** Junilla K. Larsen

**Affiliations:** Behavioural Science Institute, Radboud University, P.O. Box 9104, 6500 HE Nijmegen, The Netherlands; junilla.larsen@ru.nl; Tel.: +31-(0)-24-3612780

This Special Issue concerns the theme of how parents may influence child and adolescent weight-related and obesity developments. The ten articles in this collection cover a wide range of research designs and span a wide developmental age range of studies focusing on eating and weight-related outcomes in (very young) children or adolescents. All studies have one main central message, that is, parents, parenting and the home environment play an important role in children’s eating and weight development.

To date, two prospective studies examining eating behaviour and weight development among adolescents both suggest that parents affect later adolescents’ eating behaviour [1,2]. The study of Koning et al. [1] found that the link between parental stress and more adolescent snacking at a 1.5–2-year follow-up was mediated by the use of less autonomy supportive food parenting practices. Notably, coercive, and structured food parenting practices did not mediate this link. It might be that autonomy supportive practices are particularly important during the adolescent period, given adolescents’ emerging desire to become autonomous individuals [1]. Future longitudinal research should further examine whether and how food parenting mediates the link between parental stress and children’s snack intake and other eating behaviours in younger aged children. The other prospective study of Beijers et al. [2], including a time span of 15 years, found that lower parent–infant attachment security (i.e., using both lab and home observation methods) was associated with the increased use of emotional suppression, which was related to an increase in alexithymia, and, in turn, more emotional eating in adolescents. These links partially remained (i.e., only for the lab-based attachment measure) after controlling for parenting, suggesting that infant attachment insecurity, at least to a certain extent, may be important for the development of adolescent emotional eating beyond parenting [2]. Although this might reflect that infant attachment insecurity, specifically the type of insecurity in stress-based situations, partly forms a proxy for underlying child emotional overeating vulnerability, the links with the home-based attachment measure disappeared after controlling for parenting, also suggesting a role for early parenting preceding infant attachment and later adolescent emotional eating. Future research should further examine the many pathways that may tie parental stress to adolescents’ (emotional) overeating and obesity risk. A better understanding of these pathways is considered important, as it can inform theory and the design of better targeted preventive interventions.

In this regard, it is also important to understand contextual and individual moderators that might determine whether and how parental stress, parenting, and/or child attachment may affect children’s weight-related outcomes. For instance, Beijers et al. [2] have proposed to investigate possible sex differences in the serial mediation between attachment and emotional eating. In a large-scale cross-sectional study of more than two thousand adolescents, Dahill et al. [3] reported direct sex-specific links, whereby adolescent daughters declared themselves to be more often affected by negative weight/shape and eating comments from mothers than were sons, whereas sons perceived significantly more negative weight/shape comments from fathers than daughters. The Dahill study is one of the first examining these sex-specific correlates. Future research is needed to examine whether and how adolescent and parent gender might differentially affect the potential weight-related consequences of such negative comments, also in combination with other parenting factors.

Moreover, Quick et al. [4] found that greater family social capital (measured by supportive, engaged parenting behaviours; family cohesion; family conflict; and family meal frequency) was cross-sectionally associated with healthier home environments and weight-related (parenting) behaviours, including less coercive food parenting practices and more maternal role modeling of healthy eating and physical activity. Although Quick et al. [4] made some causal inferences that should be avoided, their results underscored the potential importance of family social capital for children’s weight-related behaviours. Future research should examine how family social capital can best be operationalized, with consistent definitions and measures that clearly separate this construct from other parenting constructs. This may advance the field and also facilitate future prospective research that might, for instance, investigate the potential interacting effects of combined family social capital and parental stress on later weight-related parenting and children’s weight-related outcomes.

Notably, some parental stress eliciting mechanisms may already operate at a very early stage, prenatal and during lactation. Larsen and Bode [5] reviewed evidence with regard to three important pathways that may explain the obesogenic programming effects of human breastmilk and, in addition, provide a research agenda for future intervention research, particularly focusing on maternal stress. They propose that early intervention efforts combining maternal stress and lifestyle, or maternal stress and parenting are particularly important in preventing excessive weight gain in both mothers and children, by attention to automatic lifestyle or parenting aspects. In a well-designed prospective study, Vinke et al. [6] found that a higher consumption frequency of SSBs among young children aged 5–6 years was strongly related to later excessive weight gain and the development of overweight at 10–11 years after controlling for baseline weight features and relevant covariates. High SSB consumption was particularly characterized by differences in consumption during main meals, rather than between meals. Younger children are probably largely dependent upon their parents for the provision of SSBs, particularly during main meals. As such, these results exposed a window of opportunity, leading to the advice for parents to offer their children sugar-free drinks to quench thirst with main meals [6]. This advice may reflect the development of changing automatic weight-related parenting, replacing context-specific unhealthy with healthy food accessibility.

Moreover, the potential importance of an automatic lifestyle may also be reflected in the data of more than four thousand six year-old children from the Generation R Study by Yang-Huang et al. [7] on the clustering of unhealthy behaviours. The results of this large-scale cross-sectional study showed that children from low educated mothers or from low-income households were more likely to be allocated in the “high screen time and physically inactive” cluster. Yang-Huang and colleagues suggest that future intervention studies may develop and evaluate programs that particularly use specific clusters of lifestyle behaviours, in order to provide more tailored support to vulnerable children and their families [7]. Future intervention studies should particularly focus on vulnerable parental populations from a lower socioeconomic-status position (SEP). However, it is rather difficult to reach and motivate these groups for such interventions. This has also been found by Harms and colleagues [8], investigating the process and impact evaluation of the family component of SuperFIT, an integrated intervention approach aiming to improve energy balance-related behaviours of young children aged 2–4 years. SuperFIT includes some important suggestions for intervention programs focusing on parents, including building attractive elements in the intervention, such as having fun and the need for peer-to-peer parent discussion, and integrating programs within existing activities, so that well-known practical barriers are avoided [8].

Another way to avoid practical barriers and reach vulnerable families may be through eHealth, offering a way to reach families with tailored health information. In this regard, Chau, and colleagues [9] found that an online pediatric-adapted liking survey (PALS) and tailored messages proved to be acceptable and useful to “vulnerable” children and parents for improving or maintaining targeted behaviours. Finally, school-based programs may also reach vulnerable children. To date, Verdonschot et al. [10] found that school-based nutrition education program effectiveness was highest in children having parents with generally lower health promotion behaviours (i.e., home-products taken to school, parent-child cooking together and talking about healthy food at home), while parents’ health promotion behaviours were positively associated with healthier child eating behaviours. As such, these findings suggest that more vulnerable children with less encouragement to eat healthily at home potentially benefit more from school-based nutrition education programs than children receiving more encouragement [10]. Future research should further examine whether parental components added to such school-based programs may also particularly benefit the most vulnerable children. However, in order for this to happen, these programs need to reach such vulnerable parents, which most often has been an obvious challenge so far, as mentioned. Future qualitative research using the Delphi method may be used to determine the most effective techniques to reach vulnerable parents within childhood obesity preventive intervention programs.

To conclude, the articles in this Special Issue support the idea that parents and the home environment play an important role in children’s weight-related development and that parental interventions to prevent childhood obesity should avoid practical barriers and facilitate the reach of vulnerable parents. Some studies refer to (clustering of) lifestyle habits, in specific home contexts (e.g., main meals), among specific vulnerable groups (e.g., lower SEP), possibly reflecting underlying automatic (parenting) processes. Other studies support the idea that parental stress, parenting, and related parent–child attachment and child emotion regulation are important factors in determining child weight-related outcomes. As parental stress may elicit a more automatic unhealthy processes within families, more attention should be paid to parental stress in childhood obesity science. Future high-quality research is needed to understand when, how and for whom parental stress begets obesity in children. Longer-term prospective designs should include more vulnerable subgroups (e.g., lower SEP) and investigate the effects of different forms of stress among diverse child age groups, while examining potential interrelated or additive mechanisms (e.g., weight-related parenting, family meal quality, child self-regulation, appetitive traits, stress responses and emotion regulation). Experience sampling methods may provide specific insight into more momentary, and potentially more automatic, mechanisms, through which parental stress may increase child obesity risk. Moreover, intervention studies targeting stress among parents may further provide causal evidence of the parental stress–child obesity link and the underlying mechanisms involved. These areas would be fruitful pursuits for childhood obesity prevention science.

## Data Availability

Not applicable.
